# New RP-HPLC Method Development and Validation for Dorzolamide in Ophthalmic Dosage Form

**DOI:** 10.1155/2018/4596141

**Published:** 2018-09-12

**Authors:** Boovizhikannan Thangabalan, Getu Kahsay, Tadele Eticha

**Affiliations:** School of Pharmacy, College of Health Sciences, Mekelle University, Mekelle, Ethiopia

## Abstract

A reversed phase liquid chromatographic method with UV detection at 254 nm for dorzolamide assay in ophthalmic solutions was developed and validated. Chromatographic separation was achieved on a Zorbax SB C_18_ (250 mm × 4.6 mm, 5 *µ*m) column kept at 30°C with an isocratic mixture of mobile phase (phosphate buffer, pH 2.5, and acetonitrile, 90 : 10 v/v) at a flow rate of 0.8 mL/min. The method was validated for its specificity, linearity, accuracy, precision, limit of detection, limit of quantification, and robustness based on ICH guidelines. The validation studies revealed satisfactory results. The proposed method has been applied for the quantification of dorzolamide in commercial samples. The developed method is fast, simple, specific, accurate, and sensitive, hence can be applied for routine quality control analysis of dorzolamide in pharmaceutical dosage form.

## 1. Introduction

**Figure 1:**
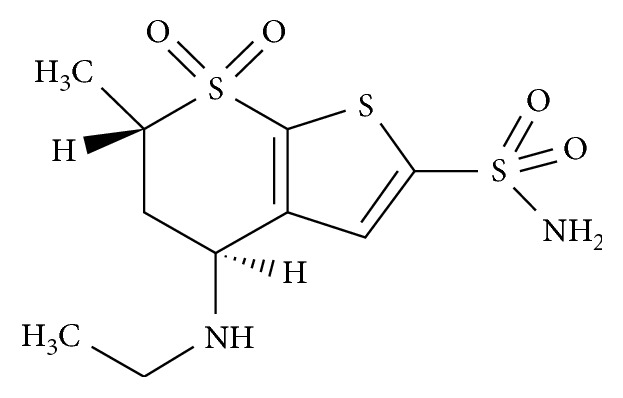
Chemical structure of dorzolamide.

Dorzolamide (DZL) hydrochloride, chemically (4*S*,6*S*)-4-(ethylamino)-6-methyl-5,6-dihydro-4*H*-thieno[2,3-b]thiopyran-2-sulfonamide 7,7-dioxide hydrochloride (Figure 1), is a carbonic anhydrase inhibitor, which is used for the treatment of glaucoma and ocular hypertension [1].

A number of analytical methods have been reported in the literature for the assay of dorzolamide individually or simultaneously with timolol maleate. These methods include reversed-phase high-performance liquid chromatography (RP-HPLC) [2–10], spectrophotometry [11–14], capillary electrophoresis [15], and others [7, 13, 16]. Both the British Pharmacopoeia [1] and United States Pharmacopoeia [17] describe liquid chromatography methods for the determination of the drug in pharmaceutical dosage forms. The present study is aimed at developing and validating a fast, sensitive, and cost-effective method for the quantification of DZL in ophthalmic dosage form.

## 2. Experimental

### 2.1. Reagents and Samples

Analytical grade potassium dihydrogen orthophosphate, ortho phosphoric acid, HPLC grade acetonitrile, and water were purchased from Merck (Mumbai, India). Pure dorzolamide active substance was obtained from Cipla Pharmaceutical Company, India.

### 2.2. Instrumentation and Chromatographic Conditions

HPLC analyses were carried out on an apparatus from Shimadzu (Japan) equipped with LC2010 series pump, Zorbax SB C_18_ (250 mm × 4.6 mm, 5 *µ*m) column, manual Rheodyne injector (with 20 *µ*L loop size), and SPD-20A UV-visible detector. Spinchrom software was used for data processing and acquisition. Sonicator (Loba, India) and pH meter (Elico, India) were employed to dissolve and/or degas the sample and measure the pH of the buffer, respectively. The mobile phase consisted of phosphate buffer (50 mM potassium phosphate, adjusted to pH 2.5 with ortho phosphoric acid) and acetonitrile in the ratio of 90 : 10 v/v. It was filtered through a 0.22 *µ*m filter (millipore filter, India), degassed in a sonicator for 10 minutes, and then pumped at a flow rate of 0.8 mL/min. The injection volume was 20 *µ*L, and the UV detection was performed at 254 nm.

### 2.3. Preparation of Reference Solution

A stock standard solution of DZL was prepared by dissolving 100 mg of pure DZL in a 100 mL volumetric flask using HPLC grade water. The drug was dissolved in 70 mL water and diluted up to the mark with the same solvent to get the solution containing 1000 *µ*g/mL of DZL.

### 2.4. Sample Preparation

From a DZL ophthalmic solution, 1 mL of the test sample was transferred into a 100 mL volumetric flask, sonicated with mobile phase for 10 minutes, and made up to the volume with the same solvent mixture. This solution was filtered through a 0.22 *µ*m filter, and 0.5 mL of the solution was diluted to 10 *μ*g/mL to get 50 *µ*g/mL with the solvent mobile phase. A 20 *μ*L aliquot was injected into the chromatographic system for analysis.

### 2.5. Method Development

The developed method was validated according to the ICH guidelines [18] for its specificity, linearity, accuracy, precision, limit of detection (LOD), limit of quantification (LOQ), and robustness.

## 3. Results and Discussion

### 3.1. Method Development and Optimization

In liquid chromatographic method development for the determination of DZL, various parameters such as detection wavelength, effect of composition of mobile phase, pH of mobile phase, flow rate, concentration of buffer solution, column temperature, and injection volume were studied and optimized. The wavelength was standardized at 254 nm based on the optimum response obtained at the specified conditions.

Several trials were performed using various composition and pH of mobile phase, flow rate, concentration of buffer solution, column temperature, and injection volume to achieve good resolution and symmetric peak shape for the drug. The optimized mobile phase consisted of phosphate buffer (50 mM potassium phosphate, adjusted to pH 2.5 with ortho phosphoric acid) and acetonitrile in the ratio of 90 : 10 v/v. Similarly, the best signal was obtained at a column temperature of 30°C, an injection volume of 20 *µ*L, and a flow rate of 0.8 mL/min reducing the run time to 7 minutes on a Zorbax SB C_18_ (250 mm × 4.6 mm, 5 *µ*m) column.

System suitability studies were conducted by injecting DZL standard solutions in six replicates and system suitability parameters such as USP plate number, 2910; tailing factor, 1.08; and retention time, 2.653 ± 0.0461 minutes (SD) were determined, which indicated satisfactory results.

### 3.2. Method Development

#### 3.2.1. Specificity

**Figure 2:**
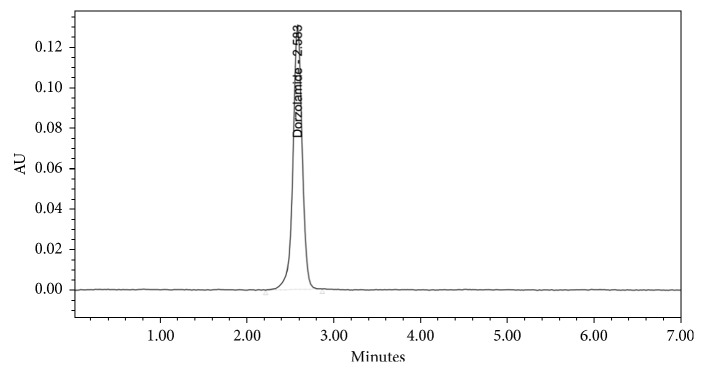
Chromatogram of dorzolamine standard solution.

**Figure 3:**
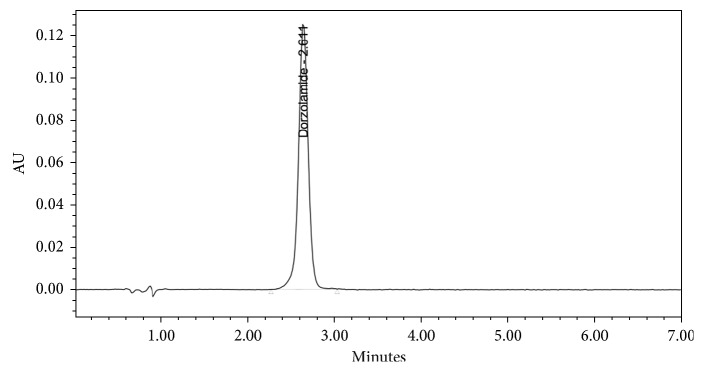
Chromatogram of dorzolamine sample solution.

**Figure 4:**
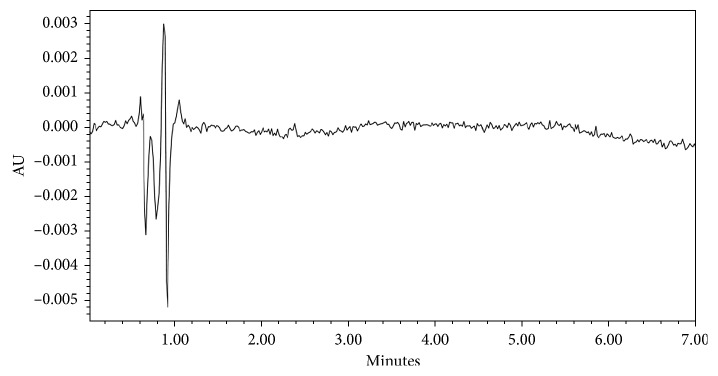
Chromatogram of placebo solution.

The specificity of the developed method was examined by injecting solutions of standard, sample, and placebo separately. The absence of interfering peaks of additives in a pharmaceutical formulation at the retention time of DZL proved the specificity of the method. Chromatograms of DZL standard, DZL sample, and placebo solutions are given in Figures 2–4, respectively.

#### 3.2.2. Linearity

Linearity was evaluated by analyzing a series of various concentrations of DZL. Six concentrations (10 , 25 , 50, 100, 125, and 150 *µ*g/mL) of DZL were injected in triplicate. Linear responses were obtained between the concentrations of the analyte and the peak areas, which was confirmed by a high correlation coefficient (*r*^2^ = 0.9999).

#### 3.2.3. Accuracy

The reliability and validity of the proposed method were examined by the standard addition technique. Known amounts of standard drug at 50%, 100%, and 150% of the test concentration were added and analyzed in triplicate. Percent recoveries ranged from 99.53% to 100.32%, which indicate the excipients in ophthalmic preparations do not interfere with DZL assay (Table 1).

#### 3.2.4. Precision

Precision of the developed method was evaluated by determining intra- and inter-day precisions as %RSD on the peak areas. The intra- and inter-day precisions were determined by analyzing the prepared samples on the same and three consecutive days, respectively, while the results of day 3 were obtained by a second analyst. The low %RSD values of the peak areas illustrate acceptable precision of the proposed methods. Findings of the precision determinations are summarized in Table 2.

#### 3.2.5. Sensitivity

The LOD and LOQ for DZL were determined based on a signal-to-noise ratio (S/N) of 3 and 10, respectively. An LOD value of 0.0405 *µ*g/mL and an LOQ value of 0.1226 *µ*g/mL were found.

#### 3.2.6. Robustness

To verify the robustness of the proposed method, the effect of small changes of relevant chromatographic parameters such as flow rate and mobile phase composition on the results was investigated. One factor at a time (OFAT) was examined sequentially and peak areas were evaluated as a response variable. The influence of flow rate at 0.7 mL/min and 0.8 mL/min and the effect of different amounts of acetonitrile (8%, 10%, and 12%) in the mobile phases were examined. The results of analysis of variance demonstrated that the peak areas were not significantly (*p* > 0.5) affected by changing these variables. Therefore, the assay values of DZL were not influenced by these small variations of the chromatographic factors investigated.

### 3.3. Application of the Method: Analysis of Real Samples

The validated method has been successfully applied to determine DZL concentrations in eye drop products. Average content of 99.92% of the label claim was obtained, which was in good agreement with the label claim for the formulation.

The developed method is more sensitive and faster than the reported analytical methods in the literature. The proposed method had lower limit of detection and quantification [4, 6, 8] and analytical run time [5]. The shorter run time leads to the low volume of mobile phase consumption, which makes the method cost-effective. Furthermore, this method is more precise than the previous method [8].

## 4. Conclusion

Although several studies have provided different methods for determination of DZL, this study provide another alternative method, which is rapid, specific, and sensitive for DZL assay in pharmaceutical dosage form. The method run time is short with excellent sensitivity: a limit of detection and quantification values of 0.0405 *µ*g/mL and 0.1226 *µ*g/mL, respectively. The developed method has been applied to ophthalmic samples.

## Figures and Tables

**Table 1:** Recovery study of dorzolamide from pharmaceutical formulation.

Formulation	Amount taken (*µ*g/mL)	Amount added (*µ*g/mL)	Amount found (*µ*g/mL)	% recovery ± SD
Eye drop	50	25	24.88	99.53 ± 0.2300
	50	50	50.14	100.28 ± 0.3534
	50	75	75.24	100.32 ± 0.2886

**Table 2:** Results of the precision study.

%RSD (*n*=6)	Day 1	0.09
	Day 2	0.10
	Day 3	0.14
%RSD (*n*=12)	Day 1-2	0.09
%RSD (*n*=18)	Day 1–3	0.11

## Data Availability

The data used to support the findings of this study are included within the article.
